# The Effects of Immediate vs Gradual Reduction in Nicotine Content of Cigarettes on Smoking Behavior: An Ecological Momentary Assessment Study

**DOI:** 10.3389/fpsyt.2022.884605

**Published:** 2022-05-11

**Authors:** Qianling Li, Xijing Chen, Xiuli Li, Monika Gorowska, Zimin Li, Yonghui Li

**Affiliations:** ^1^Chinese Academy of Sciences (CAS) Key Laboratory of Mental Health, Institute of Psychology, Beijing, China; ^2^Department of Psychology, University of Chinese Academy of Sciences, Beijing, China; ^3^YiDu Central Hospital of Weifang, Weifang, China

**Keywords:** immediate nicotine reduction, gradual nicotine reduction, craving, smoking behavior, ecological momentary assessment

## Abstract

**Background:**

In recent years, much research has examined the effects of various interventions and treatments for smoking cessation. The results suggest that interventions targeting changes of nicotine content can help smokers reduce tobacco use or quit smoking. A number of clinical studies show that smokers who received an immediate reduction in nicotine content to very low levels have significantly greater reductions in the number of cigarettes smoked and toxic substance exposure compared to those with gradual reductions. However, from the perspective of smoking craving, whether the immediate and gradual reduction in nicotine content reduce smoking by reducing cravings needs further investigation.

**Methods:**

74 eligible Participants were randomly allocated to one of the two experimental conditions: (1) immediate reduction to 0.1 mg of nicotine per cigarette (*n* = 40); (2) gradual reduction from 1.0 (0.8 g ~ 1.2 mg) to 0.1 mg of nicotine per cigarette (*n* = 34). All participants completed 1-week baseline period during which they smoked their usual cigarette, followed by 16-week of interventions. The primary outcomes included cigarette cravings and number of cigarettes smoked per day (CPD); secondary outcomes included the number of cigarette-free day and emotional states.

**Results:**

Among the 52 participants [51 (98.1%) men; mean (SD) age, 33.44 (6.71) years; mean (SD) CPD, 16.83 (9.94)] who completed the trial, significantly lower cravings for cigarettes were observed in the immediate (*n* = 25) vs. gradual nicotine reduction group (*n* = 27) in the morning (*t* = −2.072, *p* = 0.039) and after dinner (*t* = –2.056, *p* = 0.041). Compared with the baseline daily smoking, the number of cigarettes smoked per day was significantly reduced at the beginning of week 12 in the immediate nicotine reduction group (*p* = 0.001) and at week 16 in the gradual nicotine reduction group (*p* < 0.001). The number of participants with any cigarette-free day was not significantly different between the groups (*p* = 0.198). The number of cigarette-free days was significantly more in the immediate vs. gradual nicotine reduction group (*p* = 0.027).

**Conclusions:**

The significantly lower cravings were observed in the immediate vs. gradual nicotine reduction group, and led to faster reduction in the number of CPD, and a significant increase in the number of cigarette-free days. These findings add to the evidence base for reduced nicotine content in cigarettes.

**Clinical Trial Registration:**

ClinicalTrials.gov, identifier: ChiCTR2100048216.

## Introduction

Smoking remains one of the leading causes of morbidity and premature death worldwide (1–5). Long-term smoking can affect many systems of the body, resulting in serious and life-threatening diseases, such as ischemic heart disease, chronic obstructive pulmonary disease, tracheal cancer, bronchial cancer, lung cancer, stroke, etc., (5–11). According to data from the Global Burden of Disease (GBD), in 2019, the number of smokers worldwide increased to 1.1 billion, and smoking caused 7.7 million deaths worldwide. In China, the number of smokers reached 341 million (30%), and smoking causes around 2.4 million deaths a year (5). The harmful lifelong consequences of smoking lead to huge public health costs. It is estimated that smoking causes economic losses of over US $500 billion annually worldwide (12).

Nicotine is the main addictive component of cigarettes (2, 13–15). The essence of smoking addiction is nicotine dependence (16) characterized in DSM-IV, by impulsive use, discontinuation difficulty, and withdrawal symptoms after chronic use, and craving which is one of the core symptoms of nicotine addiction (17–22). Craving is common among smokers (23). Long-term use of nicotine can induce changes in the neuroplasticity of the cortex and striatum, thus forming a strong and lasting memory of nicotine addiction, resulting in a continuous craving for cigarettes (24). The existence of craving directly leads to a series of adverse consequences such as smokers' failure to quit smoking or susceptibility to relapse (25, 26). Craving is an important indicator for maintaining addictive behavior and predicting relapse after withdrawal (17, 27–35). Moreover, some studies have found that craving can significantly predict the withdrawal rate after treatment (36, 37), and therefore nicotine craving has become a criterion for estimating the effectiveness of treatment (38). Although the mechanism of craving is not completely clear, it has become an important target for the treatment of smoking addicts (39). The pain point of smoking addiction is mainly manifested in the high relapse rate, and craving is the key factor in precipitating relapse (40, 41). Thus, reducing craving has become the main target in clinical smoking cessation.

Since craving is the main precipitator of relapse, creating new intervention content that targets cravings could greatly enhance the effectiveness of the treatment. In recent years, some researchers have proposed that reducing the content of nicotine in cigarettes is an effective strategy that reduces smoking and improves public health (2, 42–48). A gradual reduction in nicotine content is a potential way to reduce the addiction to cigarettes and promote smokers to quit smoking (42, 44, 49–51). Multiple studies have shown that gradual reduction in nicotine content reduces nicotine intake, without increased exposure to tobacco toxins, and without significant “compensatory” smoking (43, 52–55). The gradual reduction in nicotine content is considered a possible smoking cessation approach (42, 44, 49–51), but it may take a long time to realize the potential health benefits (42, 56). Recent studies have found that reducing nicotine levels faster may be the same even more effective than gradual reduction (45). There is growing evidence showed that immediate reduction in nicotine content reduces the number of cigarettes smoked per day (45, 47, 57, 58), reduces exposure to toxic substances (43, 45, 52, 57–59), reduces nicotine dependence (45, 47, 57, 58), increases smoking cessation attempts (43, 45, 52, 57–59), and “compensatory” smoking is rare compared with the use of traditional nicotine cigarettes (43, 45, 52, 57, 60, 61). A comparison of the two reduction methods showed that the immediate reduction in nicotine content has a significant advantage, as it results in less exposure to toxic substances (60–64), less smoking per day (61), less nicotine dependence (56, 61), and more cigarette-free days (60, 61, 64) over time. The answer to the question of whether the gradual and immediate reduction in nicotine content reduces smoking by reducing craving is still unknown and thus has to be answered. Especially the dynamic changes in cravings shall be assessed, and therefore it is necessary to examine the relationship between daily craving changes and smoking behavior in real-time.

Ecological Momentary Assessment (EMA) is an innovative approach developed for real-time data collection, which greatly improves the field's understanding of the cognition, emotion, and behavior of smokers as they occur in the natural environment (65). The advantages of the EMA approach over retrospective self-reporting include more accurate tracking of smoking frequency and patterns, more detailed capturing of smoking cravings, and high ecological validity of the data (65–67). Since both craving and substance use are situational phenomena related to emotion and environment (68–70), measuring these variables in daily life may lead to more reliable answers. Therefore, in this study, EMA was used to assess daily craving changes and smoking behavior in real-time.

The main goal of this study was to examine the effects of the immediate and gradual reduction in nicotine content in cigarettes on cigarette craving, as well as to observe changes in smoking behavior. The main hypothesis of this research was that significantly lower cravings and lowered number of cigarettes smoked per day will be observed in the immediate vs. gradual nicotine reduction group for craving.

## Methods and Materials

This study has been approved by the Medical Ethics Committee of Shougang Hospital of Peking University and has been registered in the Chinese Clinical Trial Registry. All participants provided informed consent after they were qualified to participate.

### Study Cigarettes

To avoid the problem that offering free cigarettes increases smoking or increases the use of cigarettes with regular nicotine content, cigarettes consumed in the study were purchased by participants at designated regular tobacco companies. Study cigarettes, both menthol and non-menthol, are all of the same brand. In a study, researchers examined commercial low yield cigarettes and found that little change was seen in plasma cotinine concentration from 0.9 to the 0.4 mg nicotine yield cigarettes, suggesting compensation in smoking behavior. However, significant decreases plasma cotinine concentration and carcinogen exposure biomarker levels were observed when smokers were switched to 0.1 mg nicotine cigarettes, most likely due to the extensive filter ventilation of these “ultra-low yield” cigarettes, too much to be overcome by compensation. In addition, the 0.1 mg nicotine cigarette also produced non-significantly greater withdrawal (54). Other related studies have shown that 0.1 mg nicotine cigarettes can reduce the amount of smoking and exposure to harmful substances (52, 71, 72). Thus, the cigarette with a nicotine content of 0.1 mg was used in the immediate reduction group. Cigarettes with nicotine content of 0.6 mg (43, 53, 73–77), 0.3 mg (53, 57, 73, 75–77) and 0.1 mg (52, 71, 72) were selected in the gradual reduction group.

### Participants

Participants were recruited from Daxing District, Beijing, by handing out flyers and advertising on WeChat moments. Inclusion criteria included participants meeting the legal age for buying cigarettes (18 years old); the average daily smoking amount ≥5 cigarettes for at least 1 year; no intention to quit smoking in the past 30 days; and stable mental and psychiatric conditions. Exclusion criteria included participants who intend to quit smoking within the next 30 years; regular use of tobacco products other than cigarettes; current use of nicotine replacement or other tobacco products for cessation; symptoms of severe mental or medical illness during the past 3 months; and being pregnant or breastfeeding. A total of 94 people applied for participation, of which 74 eligible participants were included. One blinded researcher (XJC) who had no direct contact with the participants did a computer-generated randomization to assign participants to one of the two groups.

### Study Design

**Figure 1:**
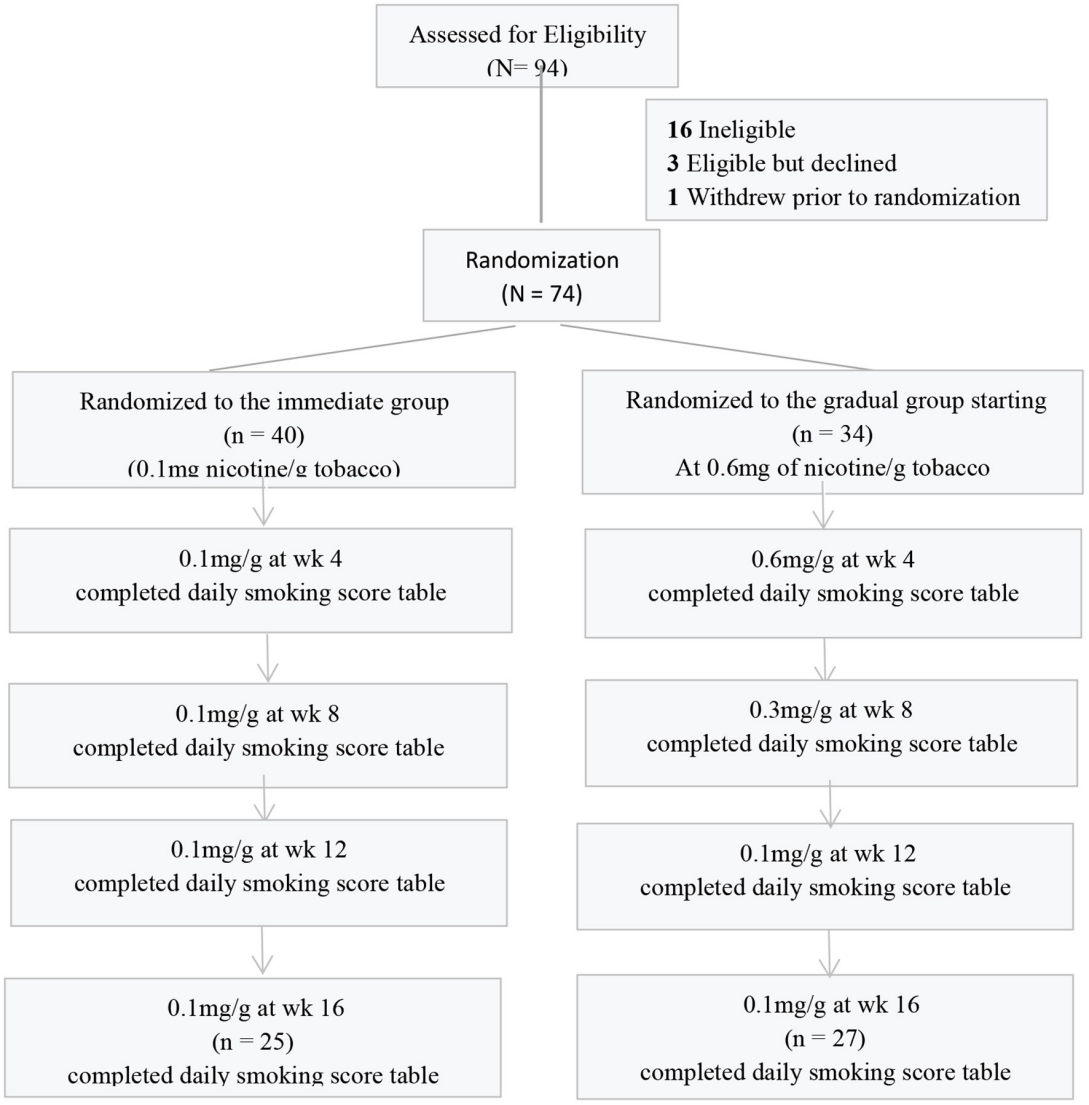
Flow chart.

This study was a randomized parallel experiment (Figure 1). Participants (*N* = 74) were randomly assigned to 1 of 2 experimental conditions: (1) immediate reduction to 0.1 mg of nicotine per gram of tobacco cigarettes (*n* = 40); (2) gradual reduction from 1.0 (0.8 ~ 1.2 mg) to 0.1 mg of nicotine per gram of tobacco cigarettes (*n* = 34).

### Procedure

Cigarette smokers were contacted by the researcher and were screened for eligibility over the telephone. Participants were told that the goal of the study was to examine how changes in nicotine content in cigarettes affect smoking behavior over time. They were also told that if they participate in the research, they would need to buy their own cigarettes during the course of the study. Eligible participants completed a baseline period of 1 week and then were randomly assigned to 1 of 2 experimental conditions for 16 weeks. Participants smoked their usual brand of cigarettes during the baseline period and used the cigarettes they were assigned to purchase during the 16-week experimental phase. In the immediate reduction group, the participants were required to smoke 0.1 mg of nicotine per gram of tobacco cigarettes for 16 weeks. In the gradual reduction group, the participants were asked to reduce the nicotine intake once every 4 weeks (0.6 mg of nicotine per gram of tobacco cigarettes were used from week 1 to week 4; 0.3 mg of nicotine per gram of tobacco cigarettes were used from week 4 to week 8; and 0.1 mg of nicotine per gram of tobacco cigarettes were used from week 8 to week 16). During the experiment, participants were told to use the designated brands of cigarettes (Study cigarettes) and try not to use their usual brand of cigarettes (Non-study cigarettes). If both types of cigarettes are used, record them and inform the researchers. Participants were required to buy the designated cigarettes during the experiment to avoid the problem that providing free cigarettes would increase smoking or use of more cigarettes with regular nicotine content. At the end of the experiment, participants were paid according to their compliance with participating in the experiment, everyone was paid for ¥200—¥400.

### EMA Assessment

During the baseline and experimental periods, this study used a score table to record the emotional state and craving degree of the participants before and after smoking in the morning and evening. The scoring method was 1–9 points to record emotional state (78), when the degree of craving was assessed, 1 indicated not wanting to smoke at all, and 9 indicated being very eager to smoke (Table 1). At the same time, the number of cigarettes smoked per day (study cigarettes and non-study cigarettes) was recorded on the score table.

**Table 1:** Daily smoking score table.

**The number of non-study cigarettes**	**The number of study cigarettes**	**Time**	**Emotion**		**Craving degree**	
			**Before smoking**	**After smoking**	**Before smoking**	**After smoking**
		Morning				
		Evening				

All participants were asked to put a score table in the cigarette packs (Table 1). In the morning when they have their first cigarette, they were asked to timely estimate their emotional state and craving values before and after smoking; in the evening, when smoking their last cigarette, participants were requested to timely estimate their emotional state and craving values before and after smoking and write down the number of cigarettes they smoked that day. The following three ways were used to provide feedback on the daily smoking to the research staff. The first one was to directly send the information on the score table to the staff through WeChat or SMS on the same day; the second one required the information on the score table to be filled into the questionnaire and submitted to the staff before going to bed every night. The third one required filling in the information on their own score table in an Excel file every day and sending it to the staff regularly.

### Questionnaire Assessment

The following measures were taken at the baseline and after the intervention: Fagerström Test for Nicotine Dependence (FTND) (57), WHO Quality of Life-BREF (WHOQOL-BREF) (79); Self Rating Anxiety Scale (SAS) (80); Self-rating Depression Scale (SDS) (81). Profile of Mood States (POMS) (82). Demographic data and smoking history were collected at baseline.

FTND: A total of 6 items, and the score of the scale ranges from 0 to 10, with higher values indicating greater dependence. The degree of nicotine dependence can be divided into five levels: very low dependence (0–2), low dependence (3–4), medium dependence (5), high dependence (6–7), and very high dependence (8–10).

WHOQOL-BREF: An international scale developed by the World Health Organization to measure an individual's health-related quality of life. 5-point scale was used to measure the quality of life from four aspects: physical health, psychological, social relationships and environment. The higher the score in each area, the better the quality of life, and the most likely area scores are 35 (physical health), 30 (psychological), 15 (social relationships) and 40 (environment).

SAS: It is used to evaluate the anxiety symptoms of adults. Compiled by W.K.Zung in 1971, there were 20 items and four grades.

SDS: It is used to evaluate the depressive symptoms of adults. Compiled by WilliamW.K.Zung in 1965, with 20 items and four grades. The severity of depression was measured from 4 aspects: psycho-emotional symptoms (2 items), somatic disorders (8 items), depressive psychological disorders (8 items) and psychomotor disorders (2 items).

POMS: The Chinese Profile of Mood States (POMS) revised by Zhu Beili (83) contains 40 items with a grade of 5. It contains seven dimensions: tension, anger, fatigue, depression, energy, panic and self-related emotions. The reliability is between 0.60 and 0.82.

### Statistical Analysis

SPSS 21.0 data analysis software was used for statistical analysis. The primary end points of this study need to be evaluated continuously every day, and the intervention time is as long as 16 weeks. Most of the dropouts dropped out of the experiment in the first few days of the intervention period, and the real-time data of the primary end points were greatly missing, and there was no late follow-up data. Thus, the analysis method used in this study is per-protocol (PP) analysis, that is, participants with good compliance and completion of the study were analyzed. Chi-square test and independent-sample *T*-test were used to analyze the differences demographic characteristics of the two groups of participants. A Chi-square test was used to compare the completion rates at week 16. Changes in cigarette craving and emotion were analyzed using a generalized linear mixed model, and the number of cigarettes was analyzed using repeated-measures analysis of variance. A Chi-square test and negative binomial regression analysis were used to analyze the number of participants with any cigarette-free day, the number of cigarette-free days among all participants. Subjective reports were analyzed by an independent sample *T*-test.

## Results

### Demographic Characteristics and Smoking History

A total of 74 participants (40 in the immediate reduction group and 34 in the gradual reduction group) were eligible, and 52 participants completed the experiment (25 in the immediate reduction group and 27 in the gradual reduction group). The completion rate of the immediate reduction group was 62.5%, and the completion rate of the gradual reduction group was 79.4%. The dropout rate of the immediate group is 37.5% (*n* = 15), and that of the gradual group is 20.6% (*n* = 7). In the later stage of follow-up, the dropout of participants in the immediate group was mainly due to the poor adaptability of some participants to very low nicotine cigarettes, and adverse events such as dizziness and nausea occurred in the first few days of using very low nicotine cigarettes, resulting in negative emotions of participants, which resulted in participants quit the intervention. The gradual group being resistant to changing cigarettes during the nicotine content change from 0.6 mg to 0.3 mg nicotine cigarettes, resulting in more dropout.

Table 2 shows the demographics and smoking history of the two groups. There is no significant difference between the two groups in demographics and smoking history, indicating that the participants in the two groups were similar (Table 2).

**Table 2:** Demographics and smoking history.

	**Immediate reduction group** ***n =* 25 (%)**	**Gradual reduction group** ***n =* 27 (%)**	**χ^2^**	***p***
Male	25 (100)	26 (96.3)	0.944	0.331
Married	21 (84)	21 (78)	0.324	0.569
**Education**
≤ High school	7 (28)	10 (37)	0.482	0.488
>High school	18 (72)	17 (63)		
	**M (** ***SD*** **)**	**M (** ***SD*** **)**	***F***	***p***
Age	34.48 (5.77)	32.48 (7.46)	0.560	0.288
Cigarettes per day	15.88 (10.89)	17.7 (9.09)	1.693	0.514
Years of regular smoking	14.72 (7.22)	13.74 (7.58)	0.000	0.634
FTND^a^	3 (2.68)	3.81 (2.68)	0.230	0.278
**WHOQOL-BREF** ^**b**^
Physical health	56.71 (10.07)	53.17 (11.31)	1.833	0.240
Psychological	55.5 (11.52)	57.72 (12.27)	0.029	0.506
Social relationships	58.67 (14.92)	58.33 (14.80)	0.062	0.936
Environment	52.75 (9.65)	55.90 (13.04)	2.260	0.330
Brief POMS^c^	111.36 (16.51)	107.37 (15.99)	0.326	0.380
SAS^d^	38.35 (7.68)	42.04 (8.23)	0.041	0.102
SDS^e^	52 (9.56)	53.47 (8.10)	0.186	0.551

a *The FTND scale ranges from 0 to 10, with higher scores indicating greater nicotine dependence*.

b *The WHOQOL-BREF produces scores for four domains related to quality of life: physical health, psychological, social relationships and environment, with higher scores indicating better the quality of life*.

c *The Brief POMS, with higher total of emotional disturb indicating a more negative emotional state, that is, a more disturb, upset or dysfunctional mood*.

d *The SAS includes 20 items, with higher scores indicating greater anxiety level*.

e *The SDS includes 20 items, with higher scores indicating greater depression level*.

### Smoking Cravings and Emotional Change in the Immediate Reduction vs. Gradual Reduction Group

**Figure 2:**
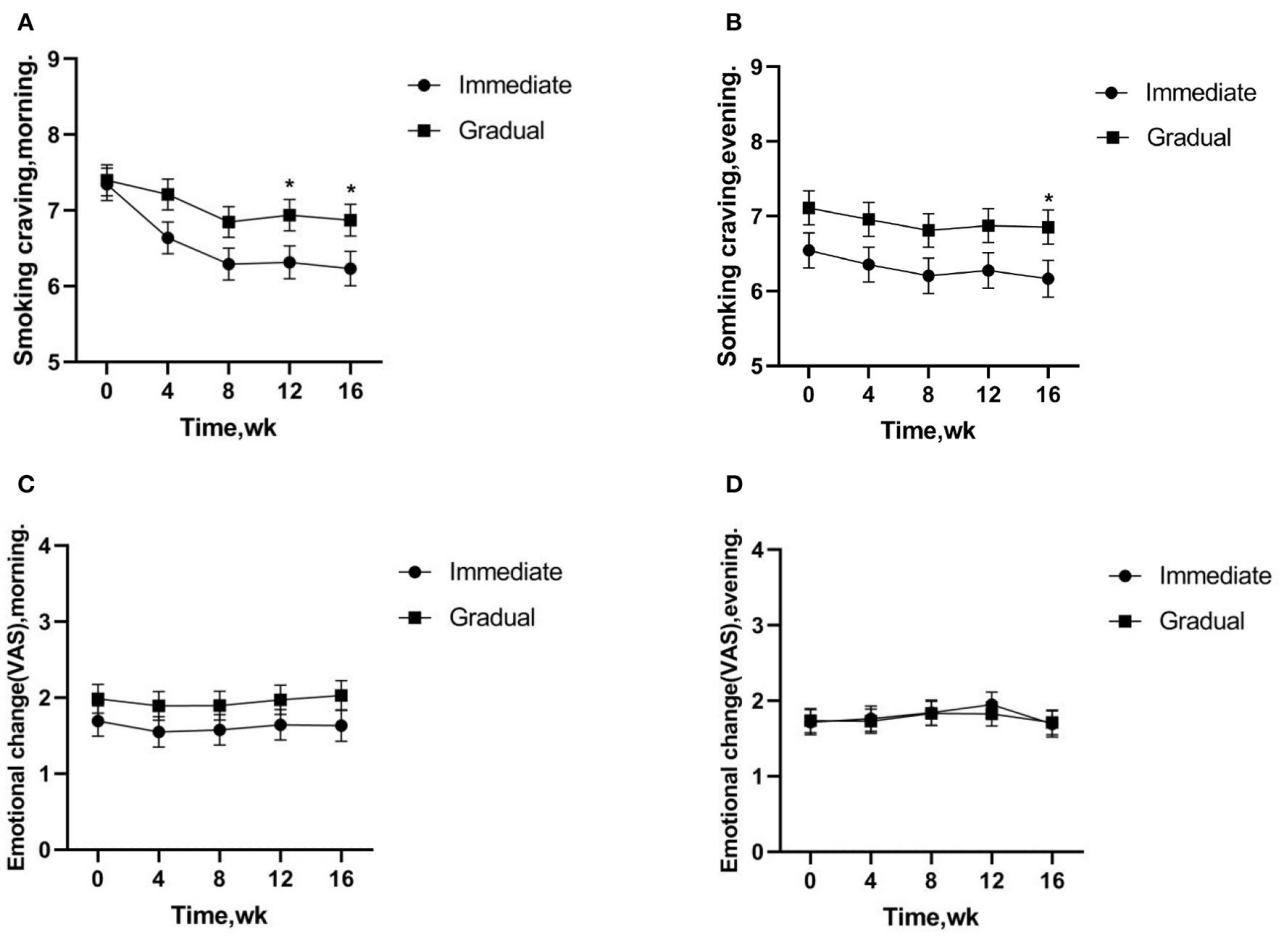
**(A,B)** Smoking cravings indicate changes in cravings before-after smoking after getting up in the morning and after dinner, respectively. **(A,B)** Significantly lower cravings were observed in the immediate vs. gradual nicotine reduction group. **(C,D)** The emotional changes of smoking indicate changes in cravings before-after smoking after getting up in the morning and after dinner, **(C,D)** there was no significant difference between the immediate reduction group and the gradual reduction group. *p < 0.05.

The changes of cigarette craving after getting up in the morning and after dinner (before smoking-after smoking) were analyzed by generalized linear mixed model. The results showed that the smoking cravings after getting up in the morning were significantly lower in the immediate vs. gradual nicotine reduction group (12 week, *t* = –2.091, *p* = 0.038; 16 week, *t* = –2.072, *p* = 0.039) after 12 weeks of intervention; and the smoking cravings after dinner were significantly lower in the immediate vs. gradual nicotine reduction group (16 week, *t* = –2.056, *p* = 0.041) after 16 weeks of intervention. Smoking cravings were significantly lower in the immediate reduction group at 4 week (*t* = 5.789, *p* < 0.001), 8 week (*t* = 6.386, *p* < 0.001), 12 week (*t* = 5.227, *p* < 0.001), and 16 week (*t* = 4.861, *p* < 0.001) of intervention vs. baseline smoking cravings after getting up in the morning. Smoking cravings were not significantly different in the gradual reduction group at 4 week (*t* = 1.593, *p* = 0.112) of intervention vs. baseline smoking cravings after getting up in the morning, but were significantly reduced at 8 week (*t* = 3.440, *p* = 0.001), 12 week (*t* = 2.442, *p* = 0.015), and 16 week (*t* = 2.464, *p* = 0.014) of intervention. Smoking cravings were significantly lower in the immediate reduction group at 8 week (*t* = 2.385, *p* = 0.018) of intervention vs. baseline smoking cravings after dinner, but no significant difference were observed at other time periods (4 week, *t* = 1.837, *p* = 0.068; 12 week, *t* = 1.559, *p* = 0.120; 16 week, *t* = 1.865, *p* = 0.063). Smoking cravings were significantly lower in the gradual reduction group at 8 week (*t* = 2.183, *p* = 0.030) of intervention vs. baseline smoking cravings after dinner, but no significant difference were observed at other time periods (4 week, *t* = 1.545, *p* = 0.124; 12 week, *t* = 1.435, *p* = 0.153; 16 week, *t* = 1.358, *p* = 0.0176) (Figures 2A,B).

The emotional changes of smoking after getting up in the morning (before-after smoking) were analyzed. The results showed that there was no significant difference between the immediate reduction group and the gradual reduction group (*t* = –1.285, *p* = 0.200). The emotional changes of smoking after dinner (before-after smoking) were analyzed. The results showed a similar effect pattern (*t* = 0.121, *p* = 0.904) (Figures 2C,D).

### Total Number Cigarettes per Day (CPD) Change in the Immediate Reduction vs. Gradual Reduction Group

**Figure 3:**
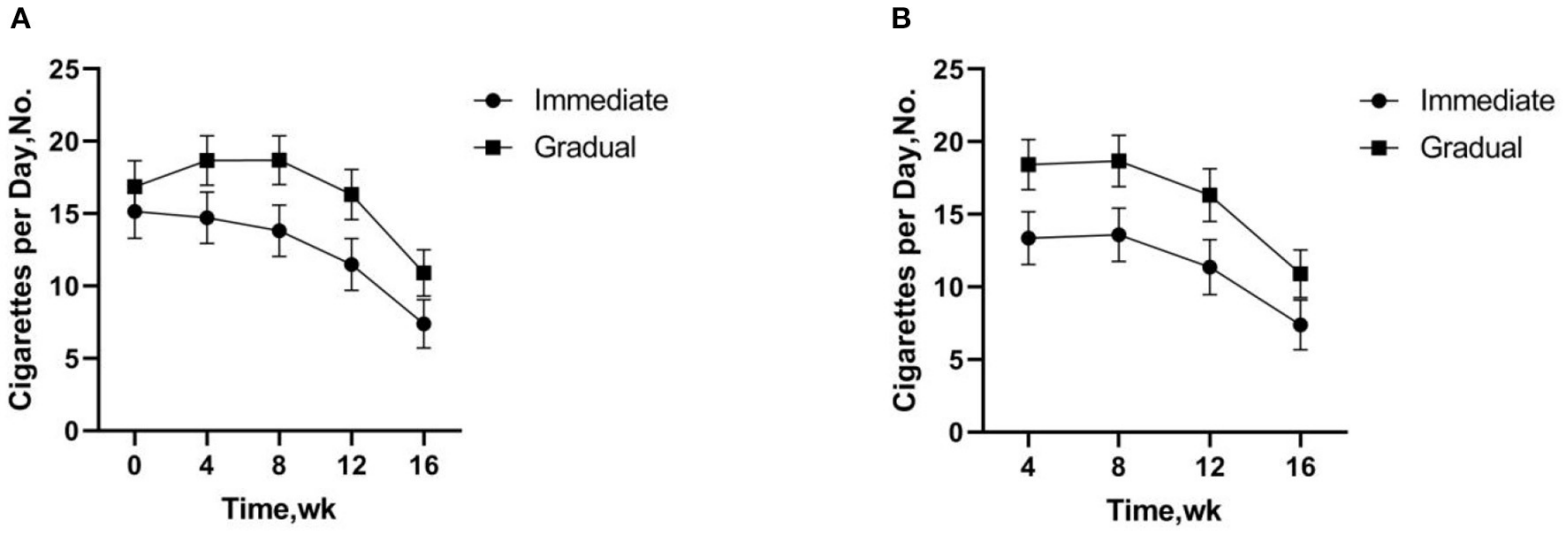
**(A)** Total cigarettes per day includes study cigarettes and non-study cigarettes. **(B)** Study Cigarettes are low nicotine content cigarettes used in the experiment. **(A,B)** There was no significant difference in the number of cigarettes per day between the immediate and gradual reduction group, but the number of cigarettes per day fewer more quickly in the immediate reduction group compared to baseline.

Two-factor repeated measurement ANOVA was used to compare the reduction effect of different intervention groups after 16 weeks of intervention. Significantly fewer numbers of total CPD were smoked in the immediate reduction group at weeks 12 (*p* = 0.001) and 16 (*p* < 0.001) vs. baseline smoking. Significantly increased numbers of total CPD were smoked in the gradual reduction group at weeks 4 (*p* = 0.006) and 8 (*p* = 0.025) vs. baseline smoking, but significantly fewer at weeks 16 (*p* < 0.001). The same effect pattern was observed in the study of cigarettes for several weeks.The number of cigarettes significantly fewer in both the immediate (*p* < 0.001) and gradual reduction group (*p* < 0.001) at the weeks 16 (Figure 3).

### The Number of Cigarette-Free Days in the Immediate vs. Gradual Reduction Group

A Chi-square test was used to analyze the number of participants with any cigarette-free day. The number of participants with any cigarette-free day was not significantly different between the immediate vs. gradual reduction group (*p* = 0.198). Negative binomial regression analysis was used to analyze the number of cigarette-free days among all participants. The number of cigarette-free days among all participants was significantly higher in the immediate vs. gradual reduction group (*p* = 0.027) (Table 3).

**Table 3:** Cigarette-free days at week 16.

**Measures**	**Immediate**	**Gradual**	**Immediate vs. Gradual**	
	**No. (%) or Mean (SD)**	**No. (%) or Mean (SD)**	**Estimated OR/IRR (95% CI)**	***p***
Any cigarette free day during weeks 0-16, No. (%)^b^	17 (68%)	18 (66.7%)	1.06 (0.33, 3.39)	0.918
Count of cigarette free days during weeks 0-16, mean (SD)^c^	24.40 (30.22)	13.00 (20.18)	0.533 (0.30, 0.93)	0.027^a^

a *p < 0.05 for the significant difference*.

b *Odds ratio (OR) was estimated based on unadjusted analysis; no abstinence being assumed for days with missing Interactive Voice Response (IVR) data*.

c *Incidence rate ratio (IRR) was estimated based on unadjusted negative binomial regression; no abstinence being assumed for days with missing IVR data*.

### Dependence, Quality of Life and Emotional Symptoms in the Immediate vs. Gradual Reduction Group

At week 16, significantly lower FTND scores were observed in the immediate vs. gradual reduction group (4.629, *p* = 0.000). There were no significant differences between the immediate vs. the gradual nicotine reduction group in four areas of WHOQOL-BREF scores: physical health (*t* = 1.324, *p* = 0.191), psychological (*t* = –0.723, *p* = 0.473), social relationships (*t* = 0.093, *p* = 0.926), and environment (*t* = –0.966, *p* = 0.339) at week 16. Total scores of emotional disturbances as assessed by the POMS were not significantly different for the immediate vs. gradual reduction group at week 16 (*t* = 0.817, *p* = 0.418). Anxiety symptoms scores as assessed by the SAS (*t* = –1.622, *p* = 0.111) and depression symptoms scores as assessed by the SDS (*t* = –0.687, *p* = 0.495) were not significantly different for the immediate vs. gradual reduction group at week 16 (Table 4).

**Table 4:** The questionnaire measures at week 16.

**Measure**	**Immediate vs. gradual reduction group**	
	**Mean difference (95%CI)**	***p***
FTND **WHOQOL-BREF** Physical health Psychological Social relationships Environment Brief POMS SAS SDS	0.166 (0.44, 1.10) 3.95 (−2.04, 9.93) −2.37 (−8.96, 4.22) 0.38 (−7.89, 8.65) −3.13 (−9.65, 3.38) 3.65 (−5.32, 12.61) −3.44 (−7.71, 0.82) −1.66 (−6.53, 3.20)	0.000 0.191 0.473 0.926 0.339 0.418 0.111 0.495

## Discussion

The present study aimed to examine (1) smoking behavior, cravings and emotional change among smokers in their daily life using Ecological Momentary Assessment (EMA) (84–86); (2) compare the effectiveness of immediate vs. gradual reduction intervention; and (3) explore whether two different nicotine reduction methods affect smoking behavior by reducing craving from a mechanism point of view.

In this study, the immediate nicotine reduction compared with gradual nicotine reduction was associated with a faster decrease in cigarette cravings, lowered cigarette cravings, faster reduction in the CPD, and more cigarette-free days over time. However, the immediate nicotine reduction caused a higher dropout rate. The use of low nicotine cigarettes had no effect on the quality of life and emotional state of the participants.

When these 2 methods were compared in this study, the results demonstrated that with immediate nicotine reduction, the smoking craving reduction could be realized sooner than gradual nicotine reduction. Therefore, the immediate reduction method is possible to facilitate cessation of cigarettes as quickly as possible. Immediate reduction method is more effective than gradual reduction method, because nicotine immediate reduction method is more conducive to promote smokers to quit smoking, faster to achieve potential public health effects. In a large clinical trial involving 1,250 smokers from 10 academic institutions, immediate reduction in nicotine may achieve positive public health effects more quickly (61).

The results of the comparison of both approaches have shown that cigarette cravings were reduced faster and significantly in the immediate reduction group. This is consistent with other research (61, 87). However, previous studies used the smoking craving scale to assess cravings periodically during the intervention, rather than continuously assessing the dynamic changes of cravings during the intervention (61). Some studies suggested that craving is an instantaneous state that changes constantly, so it may be inaccurate to assess craving over a long period of time (88). In addition, some researchers suggest that craving is a measurable continuous state (89). Therefore, in this research, we utilized EMA, to continuously assess the dynamic changes of participants' cravings over the course of intervention. The results demonstrated that cravings were significantly lower and decreased faster in the immediate nicotine reduction group.

There was no significant difference in the number of CPD between the immediate and the gradual reduction group in the study. The results of this study are not consistent with those of previous studies (61, 64). Previous studies have shown that significantly fewer numbers of CPD in the immediate than the gradual reduction group (61, 64). One possible explanation is that the duration of this study was only 16 weeks, so there were no significant differences in smoking reduction between both interventions. Another possible explanation is that the sample size of this study was too small. Compared with the baseline, both the immediate and the gradual reduction groups were able to significantly reduce the number of CPD after 16 weeks of intervention, with a faster reduction in the number of CPD in the immediate reduction group. The results have shown that the number of CPD in the immediate reduction group was reduced during the intervention and significantly reduced at week 12, while the number of CPD in the gradual reduction group increased at weeks 4 and 8 and significantly reduced at week 16. There was a temporary increase in smoking in the gradual reduction group, possibly due to compensatory smoking in moderate nicotine cigarettes (45, 52, 53, 61).

In the comparison between the immediate vs. gradual reduction group, the results demonstrated significantly more cigarette-free days among all participants in the immediate reduction group. The results are consistent with those of Hatsukami et al. (61). Both intervention methods had no effect on the quality of life and emotional state of the participants, indicating that switching to low nicotine cigarettes may be more acceptable by participants who participated in the entire intervention.

There was a higher drop-out in the immediate group. However, the withdrawal rate was no different between the immediate vs. the gradual reduction group. Other studies have shown that immediate nicotine reduction is less satisfying (61, 87), leading to more severe withdrawal symptoms (56, 61) and a higher subjects' attrition rate (61, 87) than gradual nicotine reduction. The reason why there was no difference in compliance between the immediate and the gradual reduction group may be due to the gradual nicotine reduction group being resistant to changing cigarettes during the nicotine content change from 0.6 mg to 0.3 mg nicotine cigarettes, resulting in more subjects dropout. In this study, participants were free to choose blended / flue-cured 0.6 mg nicotine cigarettes. However, when switched to 0.3 mg nicotine cigarettes, only the blend cigarettes were available. 75.0% of the participants in this study were flue-cured cigarette users, and discomfort caused by different types of cigarettes made it easier for participants to drop out during this process.

### Limitations

This study has several limitations. Firstly, the duration of this study was only 16 weeks, and the long-term effects of the two nicotine reduction methods were uncertain (61). Secondly, there was no follow-up at the end of the study. Third, the relatively small number of participants could limit the universality of the findings (43, 52). Furthermore, the average level of education of the participants was higher, and the universality of the findings is limited. Likewise, the selectivity of cigarette types was limited, which may affect the measurement of various outcomes. Moreover, the monitoring of adverse events (any negative changes in physical or mental health) and the measurement of withdrawal reaction were not carried out during the study. Seventh, in this study, most of the participants were male and there was only one female who completed the study, the study didn't compare the effectiveness of the two methods in male and in female, future studies could further compare the effectiveness of the two methods in male and in female. In addition, in this study, there is a lack of objective indicators to measure the intervention effect, and it may not be very comprehensive and objective to evaluate the intervention effect only from the self-report. in the future study, the intervention effect can be measured from multiple perspectives, and the mode of combining physiology with self-report can be adopted. Biomarkers such as cotinine can be used for physiological indicators. As well, in terms of efficacy analysis, per-protocol (PP) analysis may overestimate the efficacy. The interpretation of the results of this study is more applicable to participants who are more compliant with low nicotine cigarettes.

## Conclusions

Among smokers, the immediate nicotine reduction group led to a faster and significant decrease in cigarette cravings, a faster reduction in the number of CPD, and a significant increase in the number of cigarette-free days among all participants in the gradual reduction group.

## Data Availability Statement

The original contributions presented in the study are included in the article/supplementary material, further inquiries can be directed to the corresponding author.

## Ethics Statement

The studies involving human participants were reviewed and approved by Medical Ethics Committee of Shougang Hospital of Peking University. The patients/participants provided their written informed consent to participate in this study.

## Author Contributions

QL and YL contributed in conceptualization and methodology. YL supervised the study design and implementation. QL designed the experiment, analyzed data, wrote the paper, and revised the article. ZL designed experiment and collected data. XC provided methodological and substantive support throughout the manuscript process. MG, XC, and XL revised the article. All authors contributed to the article and approved the submitted version.

## Funding

Funding for this study was provided by Scientific and Technological Innovation 2030-Brain Science and Brain-Inspired Technology Project (2021ZD0202100).

## Conflict of Interest

The authors declare that the research was conducted in the absence of any commercial or financial relationships that could be construed as a potential conflict of interest.

## Publisher's Note

All claims expressed in this article are solely those of the authors and do not necessarily represent those of their affiliated organizations, or those of the publisher, the editors and the reviewers. Any product that may be evaluated in this article, or claim that may be made by its manufacturer, is not guaranteed or endorsed by the publisher.
